# An immune suppressive tumor microenvironment in primary prostate cancer promotes tumor immune escape

**DOI:** 10.1371/journal.pone.0301943

**Published:** 2024-11-27

**Authors:** Angelyn Anton, Ryan Hutchinson, Christopher M. Hovens, Michael Christie, Andrew Ryan, Peter Gibbs, Anthony Costello, Justin Peters, Paul J. Neeson, Niall M. Corcoran, Ben Tran

**Affiliations:** 1 Division of personalised oncology, Walter and Eliza Hall Institute, Melbourne, Australia; 2 Royal Melbourne Hospital, Melbourne, Australia; 3 Department of Surgery, University of Melbourne, Melbourne, Australia; 4 TissuPath, Mount Waverley, Melbourne, Australia; 5 Western Health, Melbourne, Australia; 6 Epworth Healthcare, Melbourne, Australia; 7 Cancer Immunology Program, Peter MacCallum Cancer Centre, Melbourne, Australia; 8 Sir Peter MacCallum Department of Oncology, University of Melbourne, Melbourne, Australia; 9 Department of Medical Oncology, Peter MacCallum Cancer Centre, Melbourne, Australia; Southern Illinois University School of Medicine, UNITED STATES OF AMERICA

## Abstract

**Background:**

Immunotherapy has demonstrated limited activity in prostate cancer to date. This likely reflects an immune suppressive tumor microenvironment (TME), with previous studies suggesting low PD-L1 expression and a sparse immune cell infiltrate. We aimed to further characterise the immune TME in primary prostate cancer and correlate immune subset densities with clinical outcomes.

**Methods:**

Two distinct cohorts of patients treated with radical prostatectomy were identified, based on the development of biochemical recurrence (BCR), one subgroup with high International Society of Urological Pathologists (ISUP) grade group, recurrent disease and a second with low grade, non-recurrent disease. A prostate immunohistochemical (IHC) antibody cocktail was used to differentiate tumor and peritumoral benign tissue. Specific CD8+, CD4+, FoxP3+, CD20+ and CD68+ cell subsets were identified using IHC staining of consecutive slides. PD-L1 and CD8/PD-L1 dual staining were also performed. Cell subset densities were quantified within tumor and peritumoral regions. We used descriptive statistics to report cell subset densities and T-tests to compare groups by age, grade and the development of BCR. Univariable and multivariable logistic regression were used to analyse risk factors for BCR and the development of metastatic disease.

**Results:**

A total of 175 patients were included, with a median age of 63 years and median pre-operative PSA of 8.2ng/ml. BCR occurred in 115 patients (66%) and 56 (32%) developed metastatic disease. CD68+ cells were the most abundant (median 648.8/mm^2^ intratumoral, 247.6/mm^2^ peritumoral), while PD-L1+ and PD-L1/CD8+ cell density was low overall (PD-L1+ median 162.4/mm^2^ intratumoral, 141.7/mm^2^ peritumoral; PD-L1/CD8+ (median 5.52/mm^2^ intratumoral, 3.41/mm^2^ peritumoral). Overall, grade group and T-stage were independently associated with BCR and metastatic disease. Higher density of peritumoral PD-L1+ cells was an independent risk factor for BCR (OR 5.33, 95%CI 1.31–21.61, p = 0.019).Although higher densities of CD8+ and CD4+ cells were observed in higher grade group 3–5 tumors, these were not associated with the development of BCR or metastasis.

**Conclusions:**

In our cohort of prostate cancer patients who underwent radical prostatectomy, higher grade group and T-stage were independent predictors of BCR and metastasis. Despite higher grade group being associated with higher CD8+ cell density, PD-L1+ and PD-L1/CD8+ cell densities were low overall, suggesting lower T cell receptor recognition of tumor antigens. Further understanding of this phenomenon would influence development of future immunotherapeutic strategies in prostate cancer.

## 1. Introduction

Prostate cancer is the second-most common cancer among men, with over 1.1 million new cases worldwide each year [1]. Although often considered an indolent cancer, there are over 300,000 deaths from prostate cancer each year, the third highest cause of cancer death [1]. Despite the recent development of multiple life-prolonging therapeutic options for metastatic disease, treatment responses are rarely durable, and the disease remains incurable. Immune cell activation through inhibition of cell surface checkpoint proteins such as PD-1, PD-L1 or CTLA-4 have demonstrated durable responses in several tumor types [2–5]. However, only modest activity has been demonstrated in prostate cancer and durable responses are rare and limited to certain subtypes, such as those with deficient mismatch repair or high tumor PD-L1 levels [6, 7].

Unique characteristics within the prostate cancer tumor microenvironment (TME) are likely to underlie the modest activity of immunotherapy. Whilst some studies have demonstrated a sparse immune cell infiltrate within prostate cancer [8, 9], other studies have demonstrated an inflammatory TME with high proportions of macrophages and T cells within the prostate cancer stroma [10], although a ‘cold’ phenotype with low levels of activation has been observed [11]. Data from The Cancer Genome Atlas described 6 distinct immune subtypes, with prostate cancer being described as inflammatory (C3) defined by increased expression of Th17 and Th1 genes [12].

While higher densities of CD8+ T cells are typically associated with a favourable prognosis in other tumor types, higher densities of both CD8+ and CD4+ cells have been shown to be poor prognostic factors in prostate cancer [8, 13–16]. The interaction between immune cells located within the tumor and those within the surrounding peritumoral benign tissue can also influence cancer progression [17]. In prostate cancer, CD8+, CD4+ and FoxP3+ cells have been shown to be more common in malignant regions compared to benign regions [18]. The PD-1/PD-L1 immune checkpoint axis also plays an important role in cancer pathogenesis by regulating cytotoxic T-cell effector responses. It is a hallmark of T cell exhaustion and leads to immune escape [19, 20]. In advanced prostate cancer, studies have observed low levels of tumor cell PD-L1 expression [21]. This possibly contributes to the limited efficacy observed with checkpoint inhibition in prostate cancer, although other factors within the immune TME are also likely to play a role.

Therefore, greater understanding of the prostate TME will help us to better understand the modest activity of immune checkpoint inhibitors and also guide future development of immunotherapeutic strategies in prostate cancer.

Our study aimed to assess the density of selected immune cell subsets present in both intratumoral and peritumoral benign (non-tumor) regions of primary prostate cancer specimens, and to examine the association of immune cell subsets with biochemical recurrence (BCR) and the development of metastatic disease following radical prostatectomy.

## 2. Materials and methods

### 2.1 Cohort selection

Patients with invasive prostate cancer who underwent radical prostatectomy were identified from a tumor registry at two sites in Melbourne, Australia. Patients within this registry had confirmed written informed consent to the Urological Biorepository Protocol, which has been approved by the Royal Melbourne Hospital Human Research and Ethics committee (HREC/14/MH/342 approved 3^rd^ February 2015). Two cohorts were selected based on the development of BCR, including one subgroup with high International Society of Urological Pathologists (ISUP) grade group (GG), recurrent disease and a second with low-grade, non-recurrent disease. Clinico-pathological information and outcome data were recorded, including age, pre-operative PSA level, T-stage, ISUP GG, time to BCR and time to the development of metastatic disease, if applicable. Patients were recruited between 15^th^ February 2015 and 1st June 2019. Clinical data were accessed by authorised study staff only between 1^st^ June 2019 and 24^th^ December 2019 through review of medical records and data were recorded in a de-identified manner.

### 2.2 Immunohistochemistry

Archival formalin fixed paraffin embedded (FFPE) prostatectomy specimens were obtained from TissuPath Specialist Pathologists Laboratory, Mount Waverley, Victoria, Australia. Tissue sections of 4μm thickness were stained from each index tumor region and surrounding benign prostate peritumoral tissue, as identified by a specialist uro-pathologist. Immunohistochemistry (IHC) was performed using the Ventana Benchmark ULTRA autostainer. Tissue sections were deparaffinised using the onboard EZ prep solution, antigen retrieval was performed using the “Cell Conditioning 1” solution (Roche/Ventana) followed by inhibition of endogenous peroxidases. IHC was performed using a Ventana Benchmark ULTRA autostainer. Tissue sections were incubated at 36°C with Confirm anti-CD3 (2GV6) rabbit monoclonal antibody for 24 minutes, anti-CD8 (SP57) rabbit monoclonal antibody for 32 minutes, Confirm anti-CD20 (L26) rabbit monoclonal antibody for 20 minutes, Confirm anti-CD68 mouse monoclonal antibody for 16 minutes and FoxP3 (SP97) rabbit monoclonal antibody for 32 minutes with amplification for 8 minutes. Markers were selected to broadly represent the presence of T-helper cells (CD4+), cytotoxic T cells (CD8+), regulatory T cells (FoxP3+), macrophages (CD68+) and B-cells (CD20+). We also performed PD-L1 (SP263) staining, a common predictive biomarker for response to checkpoint inhibition and dual staining with PD-L1 (SP263)-CD8(SP57). The Ventana Basal Cell Cocktail consisting of 34βE12 and p63 which highlights basal cells present in benign prostate glands, was used to differentiate malignant versus non-malignant areas. Representative images are presented in S1 Fig.

Positive staining for PD-L1 and cell subsets including CD4+, CD20+, FOXP3+ and CD68+ using the Ventana Basal Cell Cocktail was visualized using the Optiview DAB IHC detection kit and CD8+ cells were visualized using the ultraView universal alkaline phosphatase red detection kit.

We assessed the density (# per mm^2^) for each cell subset in the cohort, within the tumor (intratumoral) and surrounding matched benign peritumoral regions, which were demarcated by a histopathologist. Tissue sections were scanned at x40 magnification using a Roche HT iScan whole slide scanner at a resolution of 0.46 μm/pixel, and imported into Definiens Developer XD (Definiens AG, Munich, Germany). Definiens software was used to quantitate IHC staining on immune cells. Samples where IHC quantification was not achieved due to technical problems were excluded from the analysis for a given cell subtype.

### 2.3 Statistical analysis

Descriptive statistics were used to report cell subset densities in intratumoral and peritumoral benign regions and between groups based on tumor characteristics and the development of BCR or metastatic disease. BCR was defined as a post-operative PSA level of ≥ 0.2ng/ml, or a rising PSA levels that led to a change in clinical management. Metastasis were detected by conventional imaging. Categorical variables were compared using chi square analyses and continuous variables were compared using the Mann-Whitney U Test. These statistical analyses were performed using Prism software (version 8.3.1, GraphPad Software LLC, La Jolla California, USA). Univariable and multivariable logistic regression analyses were performed to analyse the effect of variables on the development of BCR or metastasis. Variables with a p-value of <0.1 on univariable analysis were included in the multivariable model. Odds ratios (OR) and 95% confidence intervals (CI) were reported for each variable. Logistic regression was conducted using Stata/SE software (version 16.1, StataCorp LLC, Texas, USA). Results with p-values of <0.05 were considered statistically significant.

## 3. Results

Our study examined a cohort of 175 patients with prostate cancer who met eligibility criteria with available archival primary tumor specimens. After a median follow-up of 82.5 months, 115 patients (66%) had developed BCR, with a median time to recurrence of 9.0 months (Range 1.5–165.4months). Baseline characteristics for each group are recorded in Table 1. Patients who developed BCR had a higher median pre-operative PSA (13.8ng/ml vs 7.9ng/ml, p = 0.001) and were significantly more likely to have ISUP GG ≥4 disease (43% vs 5%, p<0.001).

**Table 1:** Baseline characteristics.

Characteristic	Total	BCR	No BCR	P-value
	N = 175	N = 115	N = 60	
**Median Age**	62.8 years	62.9 years	62.1 years	0.09
**Median**				
**Pre-operative PSA**	8.3ng/ml	13.8 ng/ml	7.9 ng/ml	**0.001**
**ISUP Grade Group**				
**1–2**	74 (42%)	25 (22%)	49 (82%)	**<0.001**
**3**	48 (27%)	40 (35%)	8 (13%)	
**4–5**	53 (31%)	50 (43%)	3 (5%)	
**T-stage**				
**T2**	83 (47%)	26 (23%)	57 (95%)	**<0.001**
**T3-4**	89 (51%)	86 (75%)	3 (5%)	
**unknown**	3 (2%)	3 (3%)	0	

Overall, 56 patients (32%) had developed metastatic disease. Patients who developed metastatic disease had significantly higher median pre-operative PSA (14.8ng/ml vs 10.3ng/ml, p = 0.02), were more likely to have ≥T3 (80% vs 38%; P<0.001) and ISUP GG ≥4 disease (65% vs 55%, p<0.001).

### 3.1 Median density of multiple immune subsets was significantly higher within intratumoral regions compared to peritumoral regions

Of 175 prostatectomy specimens, successful cell marker IHC analysis for all subtypes was achieved in 108 specimens (62%), including >85% staining for CD20 (100%), CD8 (90%), PD-L1 (86%) and CD8/PD-L1 (86%).

**Fig 1:**
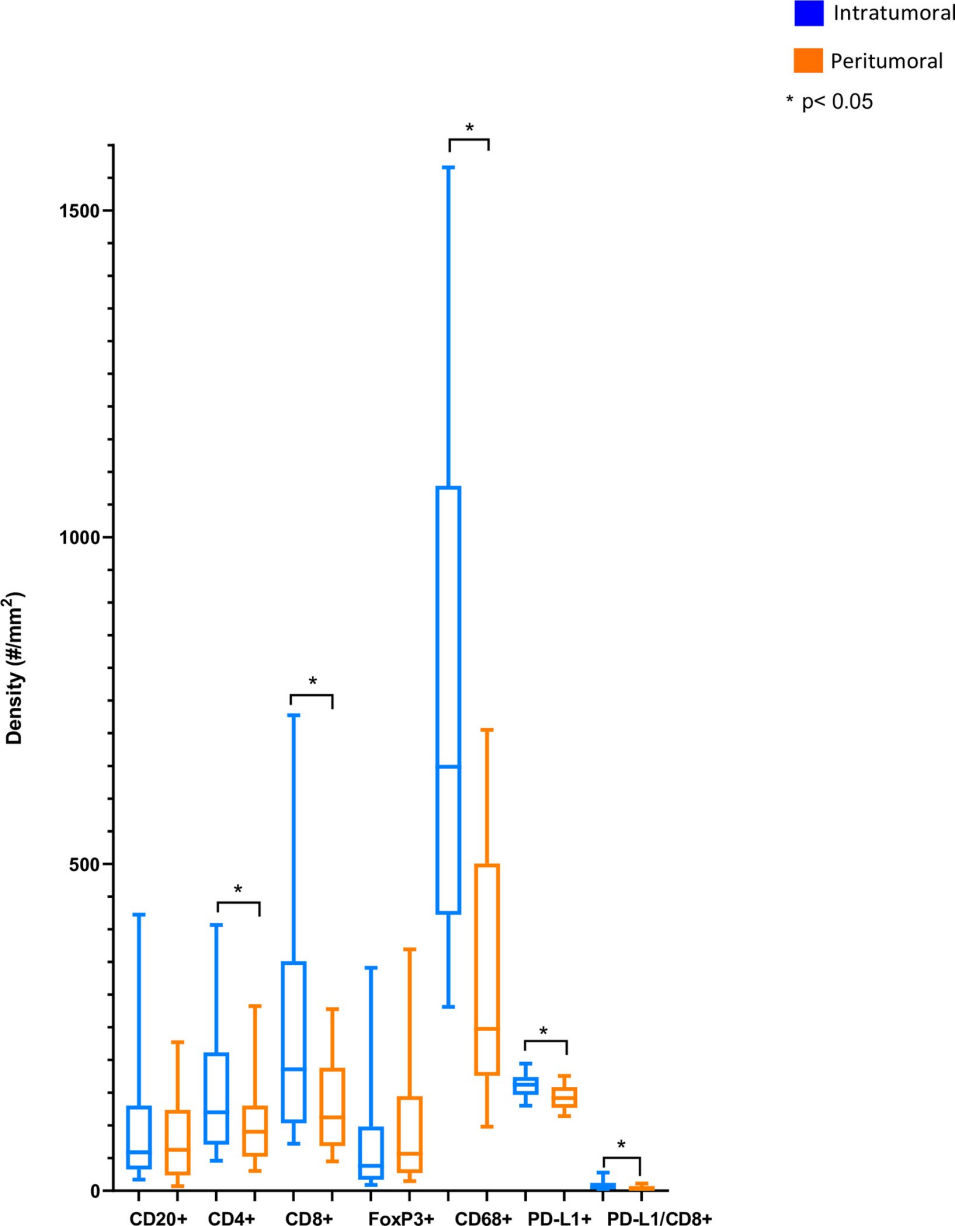
Cell subset densities in peritumoral and intratumoral regions.

To better understand the immune context of localized prostate cancer, we first evaluated immune subset density. Overall, the most abundant immune subtype was CD68+ cells (median 648.8/mm^2^ intratumoral, 247.6/mm^2^ peritumoral) followed by CD8+ cells (median 185.8/mm^2^ intratumoral, 112.4/mm^2^ peritumoral) and PD-L1+ cells (median 162.4/mm^2^ intratumoral, 141.7/mm^2^ peritumoral) was observed. The density of cells with PD-L1/CD8+ dual staining was very low (median 5.52/mm^2^ intratumoral, 3.41/mm^2^ peritumoral) (Fig 1).

We then compared the location of localized prostate cancer immune cell subsets by segmenting the tissue into intratumoral and peritumoral benign regions and quantitated each immune cell density within those histological regions. Median intratumoral density was significantly higher than peritumoral densities for multiple cell types (Fig 1), including PD-L1+ cells (p = 0.003), CD8+ cells (p<0.001), Dual CD8/PD-L1+ cells (p<0.0001), CD4+ cells (p = 0.0011) and CD68+ cells (p<0.001).

Table 2 demonstrates that the significant differences between intratumoral and peritumoral cell densities were also observed within both the cohort who developed BCR and the cohort who did not.

**Table 2:** Intratumoral and peritumoral median densities by BCR.

Cell Subtype	BCR	Peritumoral density (#/mm^2^)	p-value	Non-BCR	Peritumoral density (#/mm^2^)	p-value
	Intratumoral density (#/mm^2^)			Intratumoral density (#/mm^2^)		
**CD8+**	193.3	115.0	**<0.0001**	147.0	93.6	**0.0024**
**CD4+**	125.8	99.45	**0.007**	96.5	61.9	**0.034**
**FOXP3+**	37.6	56.5	**0.042**	51.2	61.9	0.35
**CD68+**	617.6	240.8	**<0.0001**	734.5	321.2	**0.0007**
**CD20+**	52.5	58.7	0.54	85.9	64.8	0.11
**PD-L1+**	162.0	144.4	**<0.0001**	163.7	137.4	**<0.0001**
**PD-L1/CD8+**	5.7	3.3	**<0.0001**	5.1	3.6	**0.0089**

### 3.2 Immune cell infiltrates differ between high and low GG localized prostate cancer

To investigate whether the ISUP grade influenced the density of immune infiltrating cells, we analyzed immune subset data according to ISUP category. Patients with higher ISUP GG (3–5) disease had a significantly greater density of CD8+ cells in both intratumoral and peritumoral regions within their prostate cancer specimen compared to patients with lower grade disease (intratumoral median: 207.4/mm^2^ versus 142.3/mm^2^; p = 0.01; peritumoral median: 118.0/mm^2^ vs 78.5/mm^2^; p = 0.02) as demonstrated in Table 2. Density of CD4+ cells in both intratumoral and peritumoral regions were also higher in those with GG 3–5 (intratumoral median: 138.2/ mm^2^ versus 84.8/mm^2^; p = 0.002; peritumoral median: 103.5/mm2 vs 61.7/mm^2^; p = 0.002). Median densities of all other immune cell subsets did not significantly differ between high GG (3–5) and low GG (1–2) disease, nor did dual stained PD-L1/CD8+ cells.

### 3.3 Peritumoral PD-L1+ cells were an independent predictor of BCR

On univariable analysis, higher pre-operative PSA (OR = 1.11, 95%CI 1.04–1.17, p = 0.001), ISUP GG ≥ 3 (OR = 16.11, 95%CI 7.55–34.37, p<0.001) and T-stage ≥T3a (OR = 14.07, 95%CI 6.55–30.23, p<0.001) were associated with the development of BCR (Tables 3 and 4). When examining subsets of peritumoral immune infiltrates, higher density of PD-L1+ cells (≥ median) (OR = 2.28, 95% CI 1.17–4.44, p = 0.016) was also associated with BCR. Within subsets of intratumoral immune infiltrates, a lower density of CD20+ cells (< median) was associated with BCR (OR = 0.49, 95%CI 0.26–0.91, p = 0.024) on univariable analysis but not in the multivariable model. On multivariable analysis, higher density of peritumoral PD-L1+ cells remained an independent predictor of BCR (OR 5.33, 95%CI 1.31–21.61, p = 0.019), as did higher ISUP GG ≥ 3 (OR = 10.17, 95%CI 2.55–40.59, p = 0.004) and T-stage ≥T3a (OR = 7.18, 95%CI 1.88–27.39, p = 0.001).

**Table 3:** Median cell densities by Grade Group (GG).

Cell Subset	Low GG 1–2	High GG 3–5	p-value
	#/mm^2^	#/mm^2^	
**CD8+ intratumoral**	142.3	207.4	**0.01**
**CD8+ peritumoral**	78.5	118.0	**0.02**
**CD20+ intratumoral**	60.8	57.9	0.49
**CD20+ peritumoral**	63.8	58.7	0.82
**CD4+ intratumoral**	84.8	138.2	**0.002**
**CD4+ peritumoral**	61.7	103.5	**0.002**
**FOXP3+ intratumoral**	31.7	40.15	0.22
**FOXP3+ peritumoral**	49.7	58.0	0.55
**CD68+ intratumoral**	565.1	684.8	0.50
**CD68+ peritumoral**	212.8	254.2	0.64
**PD-L1+ intratumoral**	163.4	161.0	0.75
**PD-L1+ peritumoral**	137.5	147.4	0.12
**PD-L1+/CD8+ intratumoral**	5.6	2.3	0.73
**PD-L1/CD8+ peritumoral**	3.0	3.6	0.46

**Table 4:** Univariate and multivariate logistic regression model for development of BCR.

Variable	Univariate OR	P-Value	Multivariate OR	P-Value
	(95% CI)		(95% CI)	
**Age^1^**	1.10 (1.00–1.10)	0.051	0.96 (0.87–1.06)	0.448
**ISUP Grade (Ref <3)**	16.11 (7.55–34.37)	<0.001	10.17 (2.55–40.59)	0.004
**3–5**				
**T-stage (Ref. ≤ T2c)**	14.07 (6.55–30.23)	<0.001	7.18 (1.88–27.39)	0.001
**≥T3a**				
**Pre-operative PSA^1^**	1.11 (1.04–1.17)	0.001	1.02 (0.94–1.12)	0.575
**CD20+ density (intratumoral)**	0.49 (0.26–0.91)	0.024	0.41 (0.10–1.64)	0.208
**(Ref < median)**				
**≥ median**				
**CD20+ density (peritumoral)**	0.85 (0.46–1.56)	0.598		
**(Ref < median)**				
**≥ median**				
**CD4+ density (intratumoral)**	1.33 (0.58–3.03)	0.496		
**(Ref < median)**				
**≥ median**				
**CD4+ density (peritumoral)**	1.48 (0.65–3.40)	0.351		
**(Ref < median)**				
**≥ median**				
**CD8+ density (intratumoral)**	1.83 (0.95–3.53)	0.071	1.30 (0.29–5.76)	0.728
**(Ref < median)**				
**≥ median**				
**CD8+ density (peritumoral)**	2.12 (0.90–4.96)	0.085	3.22 (0.63–16.40)	0.159
**(Ref < median)**				
**≥ median**				
**CD68+ density (intratumoral)**	1.17 (0.51–2.68)	0.704		
**(Ref < median)**				
**≥ median**				
**CD68+ density (peritumoral)**	0.89 (0.39–2.04)	0.790		
**(Ref < median)**				
**≥ median**				
**FoxP3+ density (intratumoral) (Ref < median)**	0.49 (0.20–1.16)	0.105		
**≥ median**				
**FoxP3+ density (peritumoral)**	0.57 (0.24–1.36)	0.208		
**(Ref < median)**				
**≥ median**				
**PD-L1+ density (intratumoral)**	1.21 (0.63–2.33)	0.570		
**(Ref < median)**				
**≥ median**				
**PD-L1+ density (peritumoral)**	2.28 (1.17–4.44)	0.016	5.33 (1.31–21.61)	0.019
**(Ref < median)**				
**≥ median**				
**PD-L1/CD8+ density (intratumoral) (Ref < median)**	1.51 (0.78–2.91)	0.218		
**≥ median**				
**PD-L1/CD8+ density (peritumoral)**	1.08 (0.56–2.08)	0.814		
**(Ref < median)**				
**≥ median**				

^1^ Continuous variable.

### 3.4 Peritumoral CD20+ cells were an independent predictor of metastatic disease

We hypothesised that the presence of a suppressed immune response within localised prostate cancer would be associated with reduced immune surveillance and a propensity to metastasis. In our cohort, higher density of peritumoral CD20+ cells (≥ median) (OR = 2.41, 95%CI 1.25–4.66, p = 0.009) was significantly associated with metastatic disease. In addition, we evaluated more conventional parameters of disease progression. Univariate analyses demonstrated higher pre-operative PSA (OR = 1.11, 95%CI 1.00–1.06, p = 0.031), ISUP GG ≥ 3 (OR = 9.06, 95%CI 3.79–21.67, p<0.001) and T-stage ≥T3a (OR = 7.47, 95%CI 3.42–16.30, p<0.001) (Table 5) were associated with the development of metastatic disease. Interestingly, multivariate analysis demonstrated higher density of peritumoral CD20+ cells (OR = 4.54, 95%CI 1.78–11.60, p = 0.002), ISUP GG ≥ 3 (OR = 4.98, 95%CI 1.43–17.32, p = 0.012) and T-stage ≥T3a (OR = 5.00, 95%CI 1.68–14.86, p = 0.004) as independent predictors for the development of metastatic disease.

**Table 5:** Univariate and multivariate logistic regression model for development of metastases.

Variable	Univariate OR	P-Value	Multivariate OR	P-Value
	(95% CI)		(95% CI)	
**Age^1^**	1.05 (1.00–1.10)	0.051	1.01 (0.93–1.09)	0.820
**ISUP Grade (ref <3**			4.98 (1.43–17.32)	0.012
**3–5**	9.06 (3.79–21.67)	<0.001		
**T-stage (Ref. ≤ T2c)**			5.00 (1.68–14.86)	0.004
**≥T3a**	7.47 (3.42–16.30)	<0.001		
**Pre-operative PSA^1^**	1.03 (1.00–1.06)	0.031	1.01 (0.97–1.04)	0.749
**CD20+ density (intratumoral)**				
**(Ref < median)**	0.98 (0.52–1.86)	0.958		
**≥ median**				
**CD20+ density (peritumoral)**				
**(Ref < median)**	2.41 (1.25–4.66)	0.009	4.54 (1.78–11.60)	0.002
**≥ median**				
**CD4+ density (intratumoral)**				
**(Ref < median)**	1.82 (0.87–3.82)	0.203		
**≥ median**				
**CD4+ density (peritumoral)**				
**(Ref < median)**	1.22 (0.58–2.54)	0.598		
**≥ median**				
**CD8+ density (intratumoral)**				
**(Ref < median)**	1.77 (0.91–3.45)	0.094	0.93 (0.33–2.64)	0.891
**≥ median**				
**CD8+ density (peritumoral)**				
**(Ref < median)**	1.95 (0.93–4.08)	0.077	1.07 (0.38–3.06)	0.894
**≥ median**				
**CD68+ density (intratumoral)**				
**(Ref < median)**	1.06 (0.50–2.23)	0.879		
**≥ median**				
**CD68+ density (peritumoral)**				
**(Ref < median)**	0.57 (0.27–1.22)	0.149		
**≥ median**				
**FoxP3+ density (intratumoral) (Ref < median)**	0.72 (0.33–1.57)	0.409		
**≥ median**				
**FoxP3+ density (peritumoral)**				
**(Ref < median)**	1.02 (0.47–2.20)	0.968		
**≥ median**				
**PD-L1+ density (intratumoral)**				
**(Ref < median)**	1.50 (0.76–2.99)	0.246		
**≥ median**				
**PD-L1+ density (peritumoral)**				
**(Ref < median)**	1.57 (0.79–3.12)	0.203		
**≥ median**				
**PD-L1/CD8+ density (intratumoral) (Ref < median)**	1.33 (0.67–2.64)	0.416		
**≥ median**				
**PD-L1/CD8+ density (peritumoral)**	1.18 (0.59–2.33)	0.641		
**(Ref < median)**				
**≥ median**				

^1^ Continuous variable.

## 4. Discussion

Our study examined the density of selected immune cell subsets within prostate cancer and peritumoral tissue and correlated these with the development of both BCR and metastatic disease. We demonstrated an abundant immune cell infiltrate within the prostate cancer TME, dominated by CD68+ cells, with substantial numbers of CD8+ and CD4+ T cells. In our cohort, higher peritumoral PD-L1+ cell density was an independent risk factor for BCR, while higher peritumoral CD20+ cell density was independently associated with the development of metastatic disease.

Our findings suggest that the prostate cancer TME is not an “immune dessert”. Immune cell densities within the TME in our cohort are comparable with those previously reported in melanoma, a cancer responsive to immunotherapy, with the exception of PD-L1+ cells, which were less abundant than in melanoma [22, 23]. Median CD8+ and CD4+ cell densities were higher in our cohort than those reported in colorectal cancer [24]. Like other studies, we observed a higher density of CD8+ and CD4+ cells in higher GG prostate cancer. CD8+ T cells are associated with cytotoxicity and in several cancers, are associated with superior outcomes [25–28]. In prostate cancer however, CD8+ T-cells have been documented in lower densities, are more refractory to activation and have been linked to poorer outcomes [8, 13, 29, 30]. For example, Sfanos et al. demonstrated that CD8+ prostate-infiltrating T-lymphocytes exhibited a restricted T-cell receptor repertoire [31]. A previous study also demonstrated non-functional CD8+ T cells in mice with prostate cancer, using a double transgenic model, which regained function after removal from the tumor-bearing mice, indicating a prostate-cancer specific phenomenon that leads to CD8+ T-cell inactivation [30].

Our study also demonstrated an association between higher density of peritumoral PD-L1+ cells and BCR. This is consistent with findings from another study, in which higher numbers of PD-L1+ peritumoral cells were associated with poorer clinical outcomes [32, 33]. In our study, median PD-L1+ cell density was significantly higher in intratumoral regions compared to peritumoral regions. PD-L1+ cells have been associated with poor prognosis in many tumor types, including higher risk of BCR in prostate cancer, however, correlation with long term survival remains unclear [21, 32, 34, 35]. PD-L1 expression is uncommon in metastatic prostate cancer tissue samples [21]. The reported expression in primary prostate cancer also varies significantly (7–92%) [32, 34, 36].

Tumor-infiltrating B-lymphocytes also play an important role in cancer biology but have been studied less extensively compared to T-lymphocytes [37]. In our cohort, a higher density of peritumoral CD20+ cells was an independent risk factor for development of metastatic disease. While higher density of CD20+ cells have been associated with improved prognosis in some tumor types, they have demonstrated poorer prognosis in prostate cancer, consistent with our findings [38]. Preclinical models suggest B cells promote castration-resistance through cytokine production [39] and the anti-CD20 antibody Rituximab has been shown to modulate the prostate TME [40]. Further studies are required to validate these findings, which have potentially important therapeutic implications.

Tumor associated macrophages are known to play an immune inhibitory role, differentiating from myeloid-derived suppressor cells (MDSC) and suppressing T-cells [41, 42]. In our study, CD68+ macrophages were the most abundant immune subset and were more abundant within intratumoral regions compared to peritumoral regions. This may support our hypothesis of an immune-suppressive TME, given their inhibitory potential on other immune cells. This is consistent with results from another study demonstrating the importance of monocytes and macrophages in disease recurrence and progression, based on differential transcript abundance analysis and multiplex immunohistochemistry [43]. Our results were limited by the lack of macrophage markers that specifically indicate an inhibitory function and correlate with poor clinical outcomes, particularly CD163 and VSIG4 [44]. Although beyond the scope of our study, further analysis of differential gene expression would potentially enable a more robust assessment of immune cell function and activation within our cohort and validation with an independent dataset would enable confirmation of our study findings.

Despite the limitations of our case-control cohort, which include inherent selection bias, our findings confirmed the significance of the established poor prognostic factors of higher GG and T-stage, which were independently associated with BCR and metastasis. Our study examines the TME within primary prostate cancer, which may differ from that of metastatic lesions, as demonstrated by the differences in reported PD-L1 levels [32, 34, 36]. However, metastatic biopsies are not routinely performed due to the predominance of bone-only disease and the high failure rate. Furthermore, understanding the TME in earlier disease is relevant to the development of future immunotherapeutic and immunomodulatory strategies.

## 5. Conclusions

Our study of primary prostate cancer demonstrates an abundant immune cell infiltrate dominated by CD68+ cells with higher density of peritumoral PD-L1+ cells being an independent risk factor for BCR and of peritumoral CD20+ cells being an independent risk factor for metastatic disease. Greater understanding of the immune TME, particularly mechanisms of immune cell signalling and inactivation, will provide guidance for the development of future therapeutic strategies.

## Supporting information

S1 Fig Representative images of immunohistochemistry staining and prostate tumour region detection. (TIF)

S1 Raw data (XLS)
