# Supplementary figures and images for: An immune suppressive tumor microenvironment in primary prostate cancer promotes tumor immune escape
Source: PLoS One. 2024 Nov 27;19(11):e0301943. doi: 10.1371/journal.pone.0301943 (PMC11602054; doi:10.1371/journal.pone.0301943)

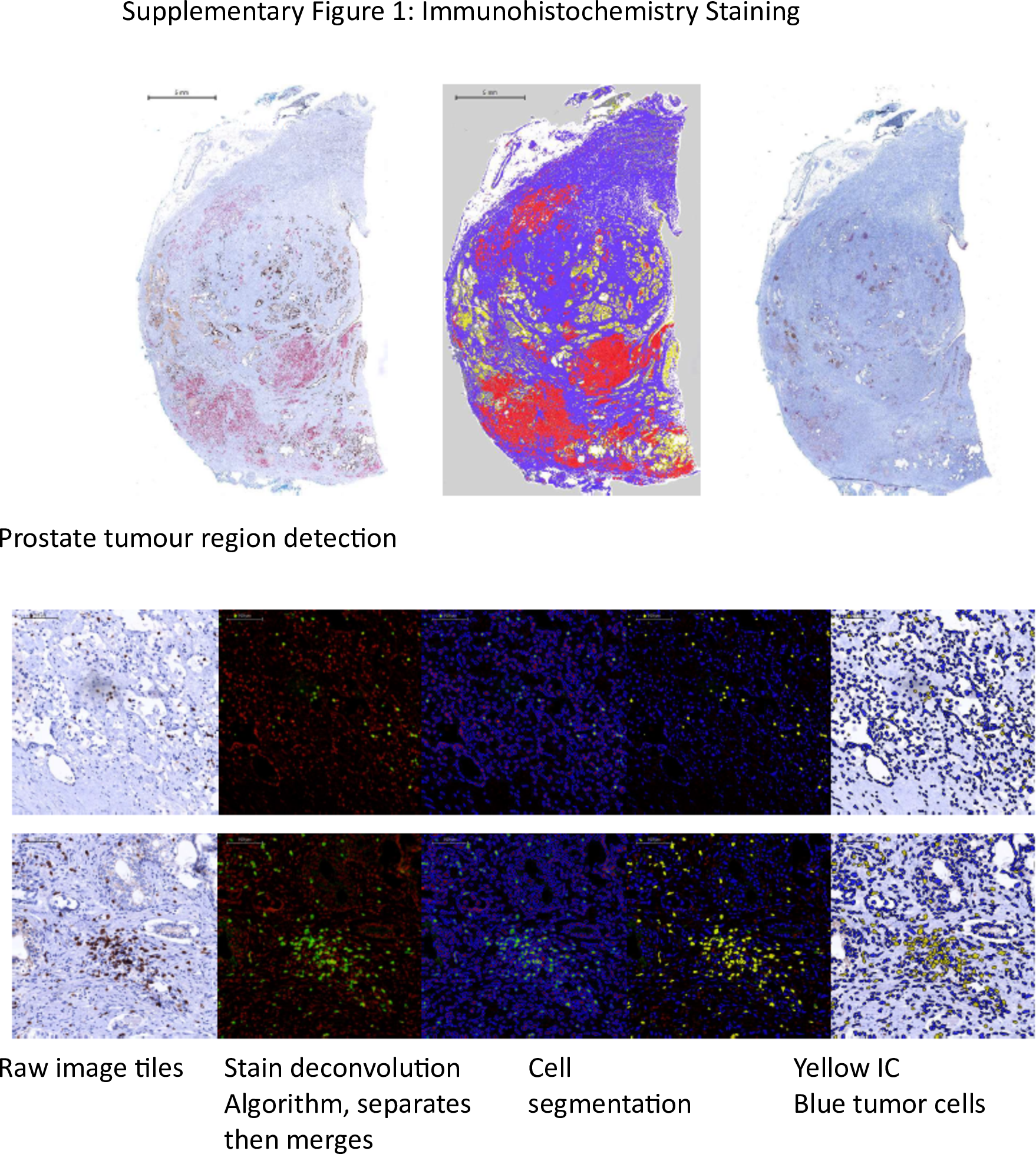

Supplement: S1 Fig — (TIF) [file pone.0301943.s001.tif]
